# Retrograde peri-implantitis

**DOI:** 10.4103/0972-124X.65444

**Published:** 2010

**Authors:** Jumshad B. Mohamed, B. Shivakumar, Sabitha Sudarsan, K. V. Arun, T. S. S. Kumar

**Affiliations:** Department of Periodontology and Oral Implantology, Sree Balaji Dental College and Hospital, Uthandi, Chennai, India; 1 Department of Periodontics and Implant Dentistry, Ragas Dental College and Hospital, Uthandi, Chennai, India

**Keywords:** Implant, retrograde peri-implantitis, regeneration

## Abstract

Retrograde peri-implantitis constitutes an important cause for implant failure. Retrograde peri-implantitis may sometimes prove difficult to identify and hence institution of early treatment may not be possible. This paper presents a report of four cases of (the implant placed developing to) retrograde peri-implantitis. Three of these implants were successfully restored to their fully functional state while one was lost due to extensive damage. The paper highlights the importance of recognizing the etiopathogenic mechanisms, preoperative assessment, and a strong postoperative maintenance protocol to avoid retrograde peri-implant inflammation.

## INTRODUCTION

During the past decade, the use of osseointegrated implants has become an increasingly important treatment modality for the replacement of missing teeth in fully and partially edentulous patients The success of osseointegrated dental implants has revolutionized dentistry.[1] With more than three decades of evidence to support the clinical use of osseointegrated dental implants, implant-related prosthesis has become a predictable method of replacing missing teeth.[2,3]

The widespread use of these implants has in recent years, produced different types of complications. Retrograde peri-implantitis, a lesion occurring at the periapical area of an osseointegrated implant, has recently been described[4] as a possible cause for dental implant failure. The etiology of “implant periapical lesion” (IPL) could be attributed to overheating of the bone;[5,6] overloading of the implant;[7] presence of a pre-existing infection or of residual root particles and foreign bodies in the bone;[8,9] implant contamination during production or during insertion[10] or placement of the implant.

The usual plaque related or occlusion related peri-implant failure is relatively easily identified when compared to the retrograde peri-implant lesions. With the ever increasing esthetic demands of patients, early loading of implants, especially in the anterior segment has become a necessity. As a number of teeth in the anterior segment are lost due to trauma and other non-periodontal causes, the clinician must be aware of the potential risk of developing retrograde peri-implantitis due to periapical pathology in the existing socket/adjacent teeth. This paper presents four cases of retrograde peri-implantits (each with different etiopathogenic mechanism), three of which were successfully managed and subsequently restored to function. The paper then discusses the possible etiopathogenic mechanism and suggests guidelines for the early identification and management of peri-implantitis.

## CASE SERIES

### Case I [Figure 1a to 1j]

**Figure 1a:**
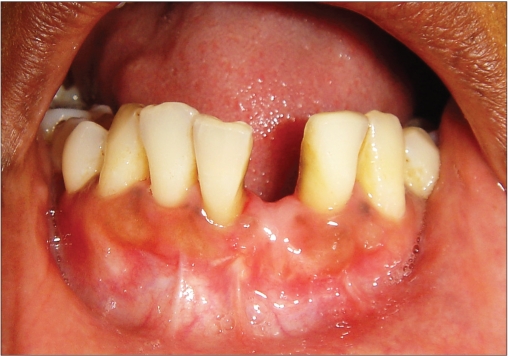
Preoperative photograph

**Figure 1b:**
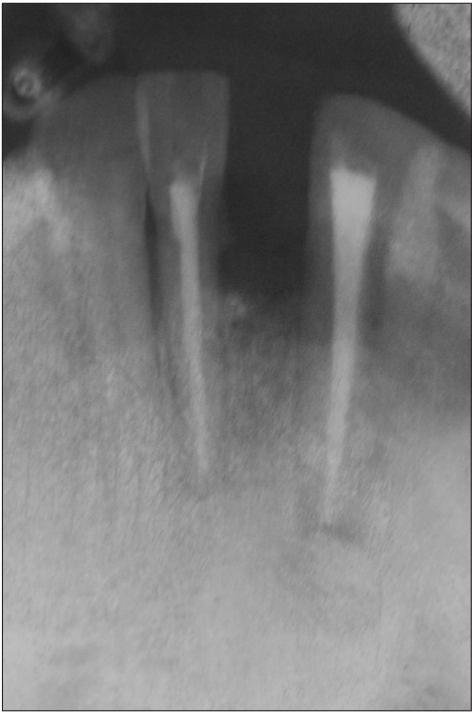
Preoperative radiograph

**Figure 1c:**
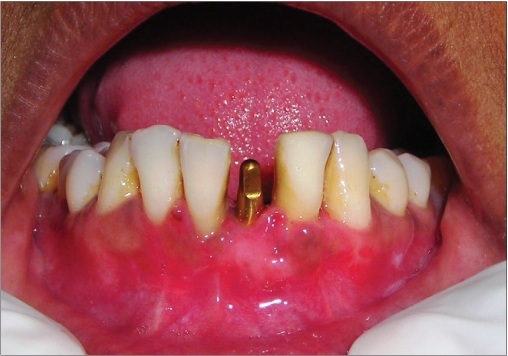
Six week photograph

**Figure 1d:**
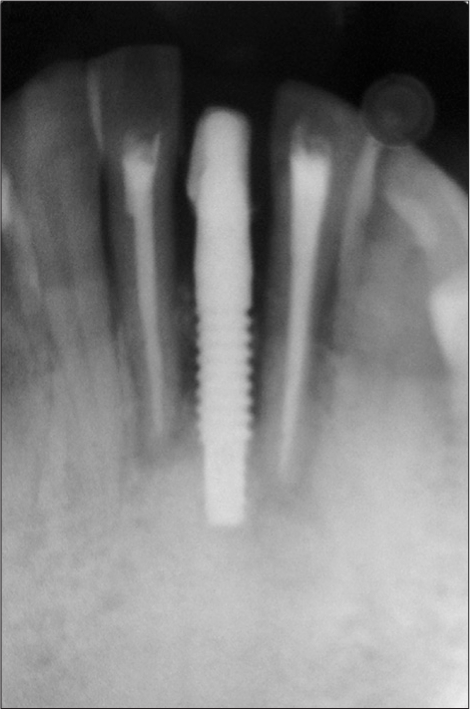
Six week radiograph

**Figure 1e:**
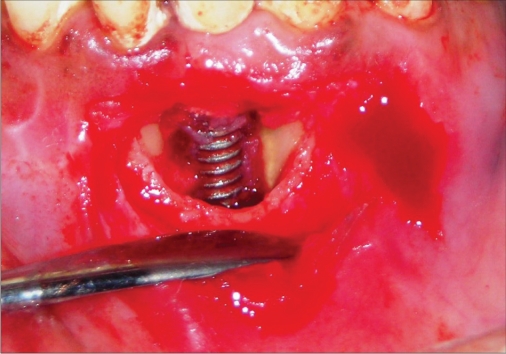
Operative photograph

**Figure 1f:**
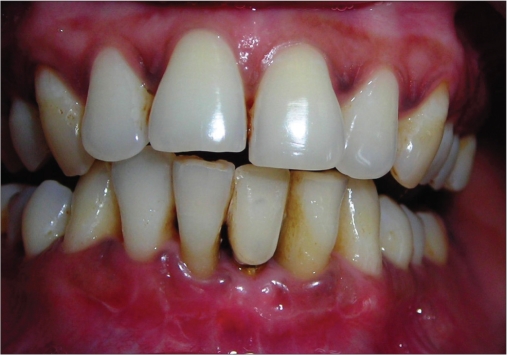
Six month photograph

**Figure 1g:**
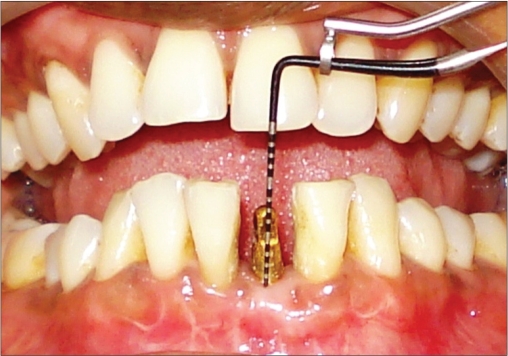
Six month probing

**Figure 1h:**
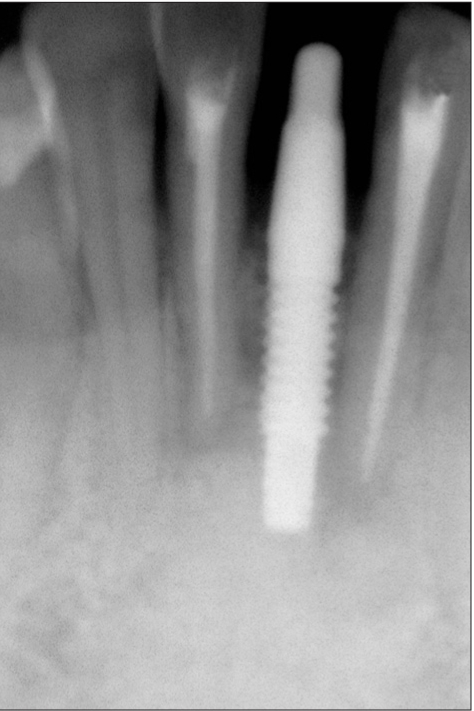
Six month radiograph

**Figure 1i:**
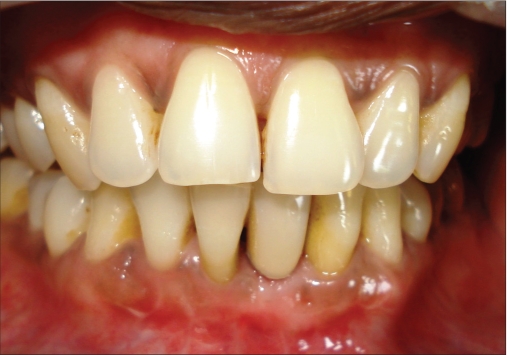
One year photograph

**Figure 1j:**
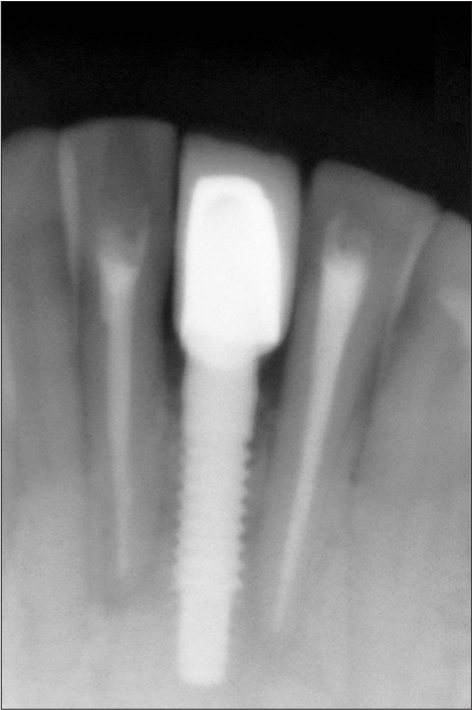
One year radiograph

A 42-year-old female patient presented with a history of failed endodontics followed by extraction and socket preservation in relation to #31 six months prior to reporting to the implant clinic. The adjacent teeth #32 and #41 showed endodontic restorations. After clinical and radiological evaluation it was decided to place a 3.0 × 12 mm single piece implant^$^. Adequate primary stability was obtained at the time of placement. When reviewed after six weeks, the site showed signs of abscess formation in the alveolar mucosa in relation to implant #31. There was no evidence of probing depth around the implant, but radiographs revealed peri-implant bone loss at the middle third region. The implant was found to be stable with no mobility. Open flap debridement was done and the implant surface decontaminated with universal implant deplaquer^$$^. The implant was subsequently followed up for a period of one year with regular three-month clinical and radiological reviews.

#### Discussion

The normal peri-implant sulcus depth and absence of other inflammatory signs in the peri-implant mucosa suggests that anterograde peri-implantits was not the cause for bone loss observed in this case. Even though endodontic therapy was performed in the adjacent teeth, placement of implant might have triggered latent periapical pathology[11,12] from the adjacent teeth. Recent evidence suggests the existence of an autoimmune response[13,14] in the periapical area in relation to an antigen which may be microbial in origin. The host response that is triggered off, may affect the host tissues as a result of similarity between the microbial and the host antigens, such as the heat shock proteins. In such instances, even after thorough debridement of the root canal has resulted in elimination of the microbial antigens, the host response may ensure continuation of an active inflammatory process.

The process of implant placement could result in activation of this latent response either due to overheating or contamination or a combination of both. This activation could have resulted in the rapid bone loss in a short period of time. The importance of periodic clinical and radiographic examination of implants that are placed adjacent to endodontically treated teeth has to be emphasized and a shorter recall program has to be instituted to identify and manage retrograde peri-implant bone loss in its early stages.

### Case II [Figure 2a to 2j]

An 18-year-old male presented with a history of trauma two weeks prior to presentation resulting in avulsion of tooth #21. After clinical and radiological evaluation a 3.0 × 15 mm single piece implant^$^ was placed with adequate primary stability. In two weeks’ time patient presented with signs of peri-implant abscess formation and mobility of the implant in relation to tooth #21. Radiograph revealed peri-implant bone loss at the apical third as well as the adjacent tooth #22. Open flap debridement was done with universal implant deplaquer^$$^ and the osseous defect was filled with calcium phosphate and Hydrase^#^. Tooth #22 was endodontically treated. The implant was subsequently followed up for a period of one year with regular three-month clinical and radiological reviews.

**Figure 2a:**
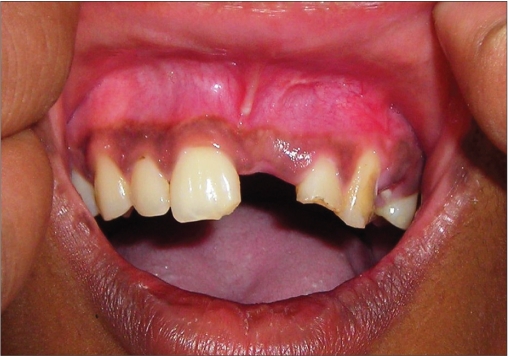
Preoperative photograph

**Figure 2b:**
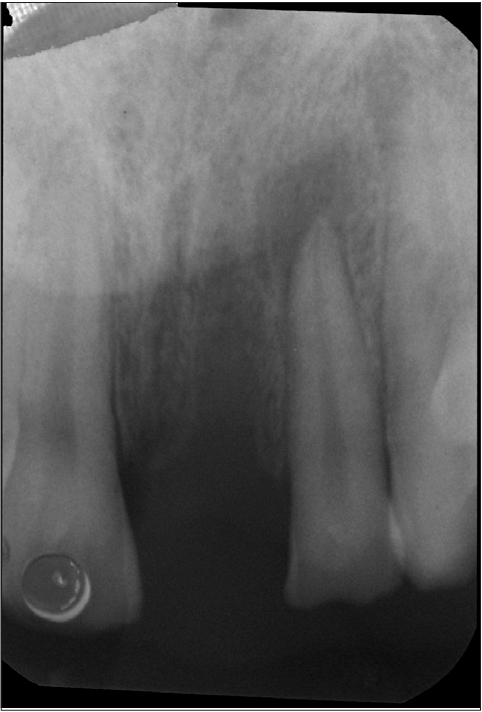
Preoperative radiograph

**Figure 2c:**
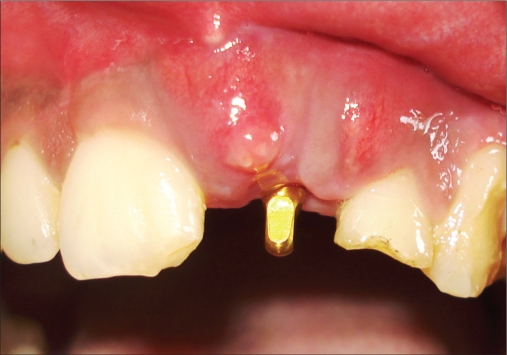
Two week photograph

**Figure 2d:**
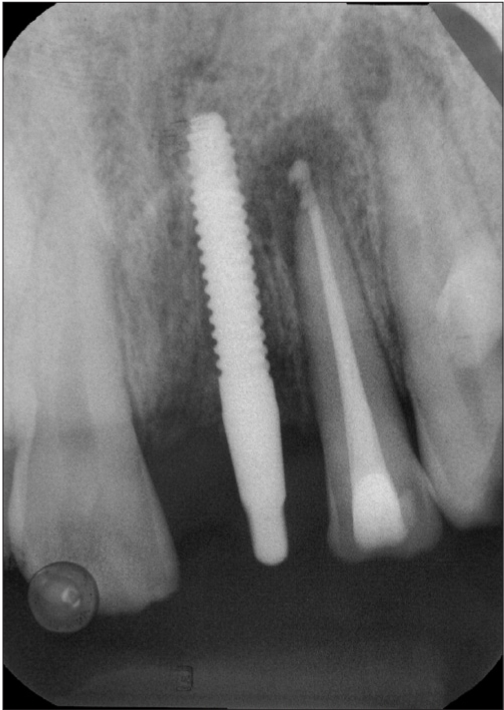
Two week radiograph

**Figure 2e:**
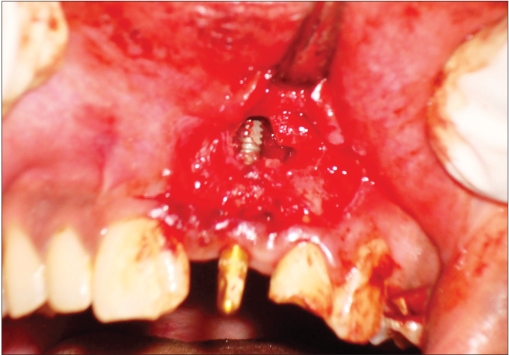
Operative photograph

**Figure 2f:**
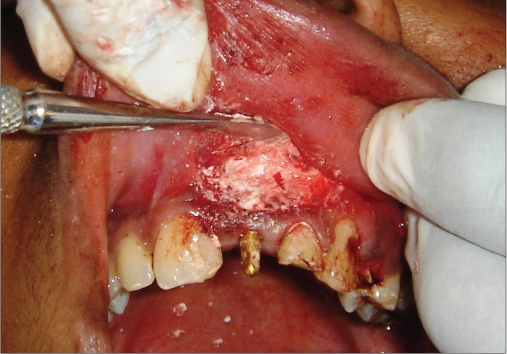
Bone graft placed

**Figure 2g:**
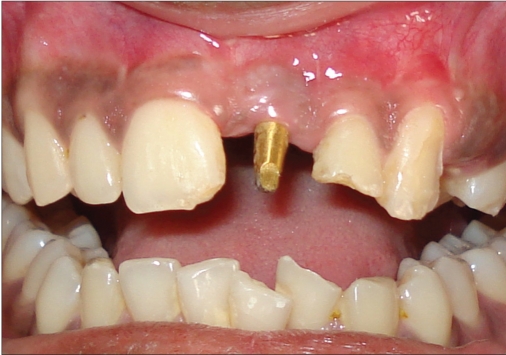
Six month radiograph

**Figure 2h:**
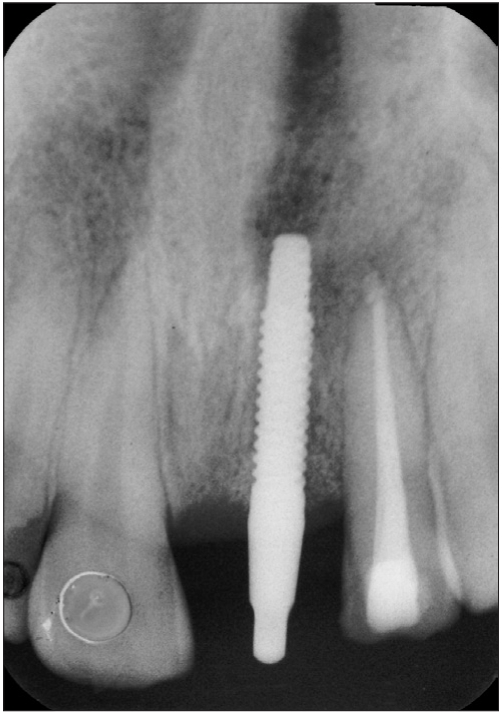
Six month radiograph

**Figure 2i:**
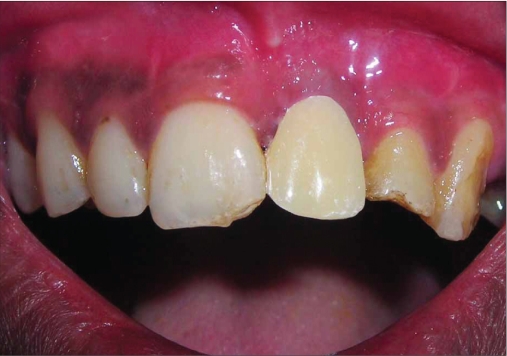
One year photograph

**Figure 2j:**
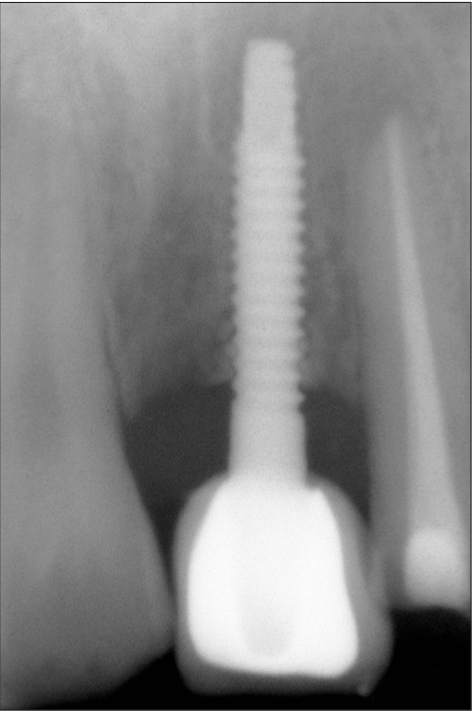
One year radiograph

#### Discussion

In this case, the implant was placed in an avulsed site adjacent to tooth #22 that exhibited signs of trauma (Ellis class II fracture). The tooth was tested for vitality using the EPT (electric pulp tester), which revealed the presence of a vital pulp. Subsequently, the implant placement was undertaken without endodontic therapy in tooth #22. This case illustrates the limitations of using the EPT (which is actually an indicator of nerve stimulation) when testing the vitality of the tooth. The undetected pericapical pathology flare following implant placement resulting in the retrograde peri-implant lesion is identified in this case. The fractured tooth adjacent to an edentulous site must be critically evaluated for evidence of dormant periapical lesions[14] and the results obtained from EPT may not be considered a gold standard for vitality of the pulp. In suspected cases where the fracture line is close to the pulp, it may be prudent to consider intentional endodontic therapy prior to implant placement regardless of the results obtained from the EPT.

Management of the peri-implantitis was performed as stated by Tözüm M *et al*,[9] and Peñarrocha Diago M *et al*,[8] by treating the periapical implant pathology and the adjacent natural tooth without the removal of the implant. The treatment procedure included root canal treatment followed by the debridement of the apical bone lesion, and guided bone regeneration. Smaller peri-implant lesions heal well even without a placement of bone replacement graft; however, in larger defects it may be prudent to place these materials to enhance healing. The placement of the graft (calcium phosphate) allowed greater chance for new bone formation as otherwise repopulation of the wound site by gingival cells, could result in a fibrous rather than osseous healing.

### Case III [Figure 3a to 3k]

**Figure 3a:**
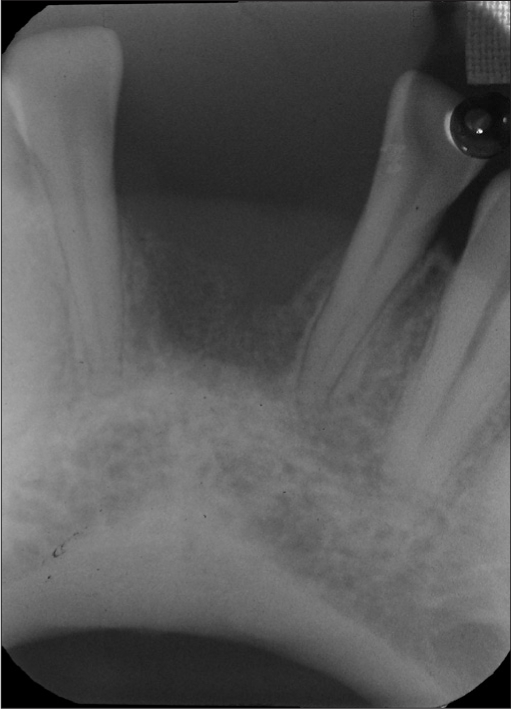
Preoperative radiograph

**Figure 3b:**
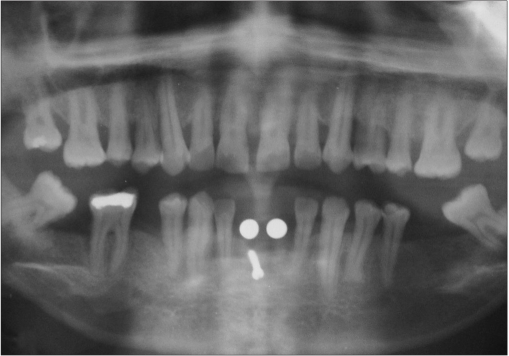
Radiograph showing block graft

**Figure 3c:**
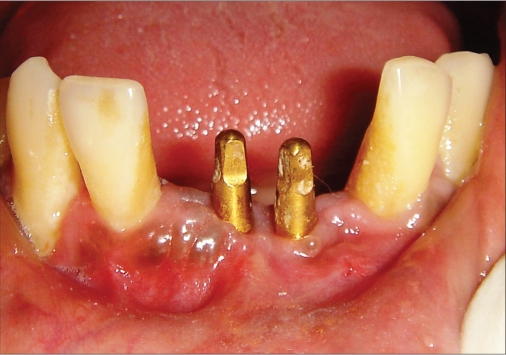
Four weeks photograph

**Figure 3d:**
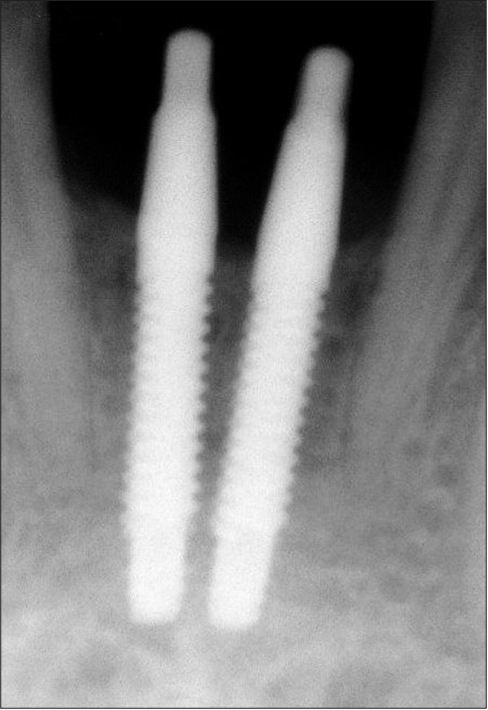
Four weeks radiograph

**Figure 3e:**
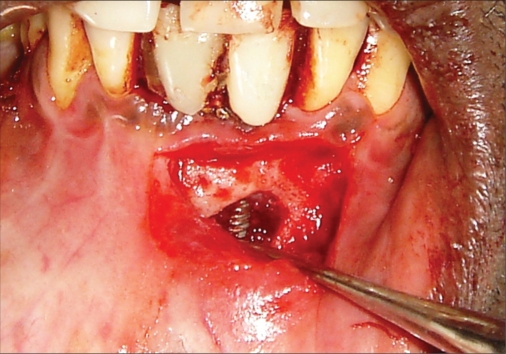
Operative photograph

**Figure 3f:**
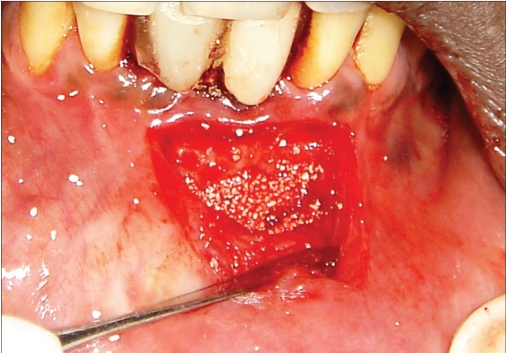
Bone graft placed

**Figure 3g:**
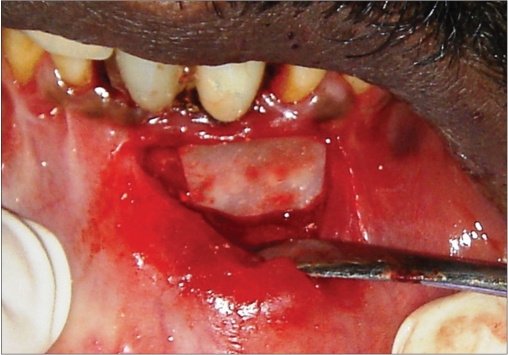
Membrane placed

**Figure 3h:**
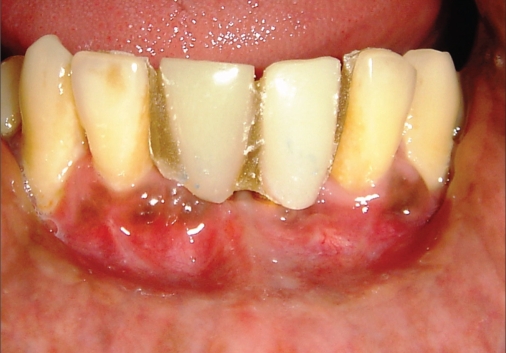
Six month photograph

**Figure 3i:**
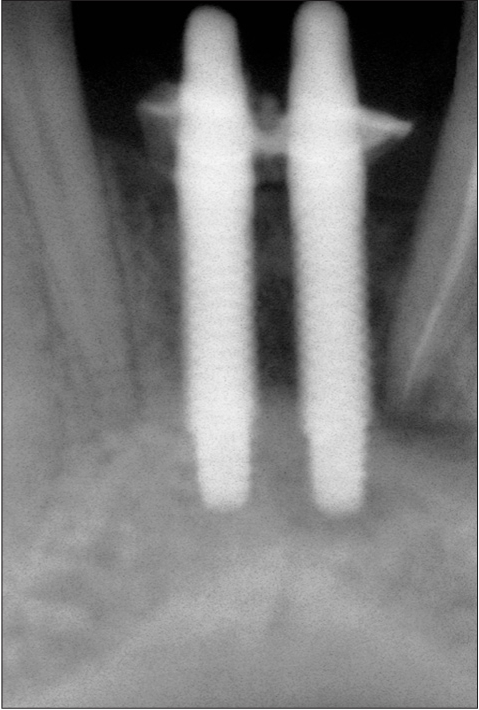
Six month radiograph

**Figure 3j:**
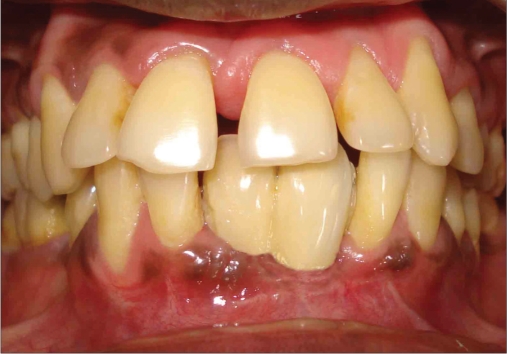
One year photograph

**Figure 3k:**
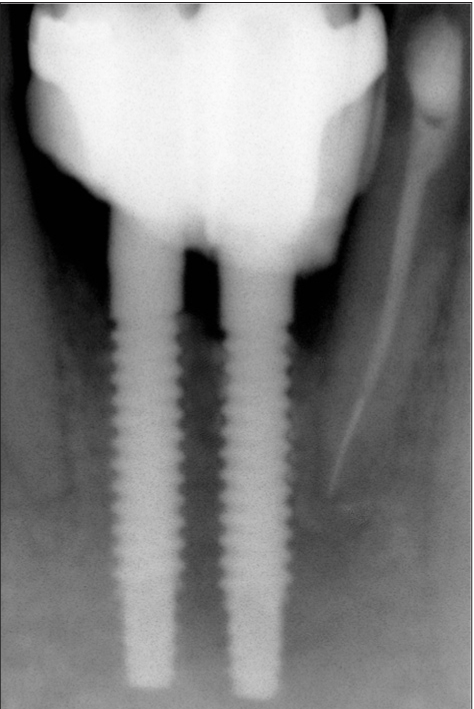
One year radiograph

A 38-year-old male presented with a history of treated chronic periodontitis and missing teeth #31 and #41. Clinical and radiological examination revealed inadequate ridge width for implant placement; hence block graft augmentation was performed with a ramus block graft at the site. After 6 months, two 3.0 × 15 mm single piece implant^$^ was placed in relation to #31 and #41 with adequate primary stability. At one-month review abscess formation was noticed around implant #31 with mobility. Implant #41 was intact with no signs of peri-implantits. Radiographic examination revealed peri-implant bone loss at the apical third as well as the adjacent tooth #32. A flap was raised and thorough debridement was done with universal implant deplaquer^$$^ followed by placement of bioceramic bone graft^##^ and collagen membrane^*^ along with endodontic restoration of tooth #32 on the same day. The implant was subsequently followed up for a period of one year with regular three-month clinical and radiological reviews.

#### Discussion

Existence of active periodontal inflammation in the adjacent teeth is one possible etiopathogenic mechanism[15] responsible for periapical pathology and subsequent spread of infection to involve peri-implant tissue in a retrograde manner. Existing periodontal disease must be scrutinized carefully to ensure resolution of the active inflammatory lesion before implant placement is undertaken. The usual clinical parameters of BOP, PD, and CAL have to be repeated serially after surgical/nonsurgical periodontal therapy to ensure stability of the periodontium prior to implant placement.

### Case IV [Figure 4a to 4f]

**Figure 4a:**
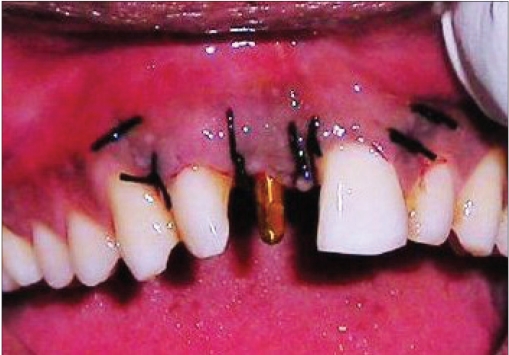
Immediate postoperative photograph

**Figure 4b:**
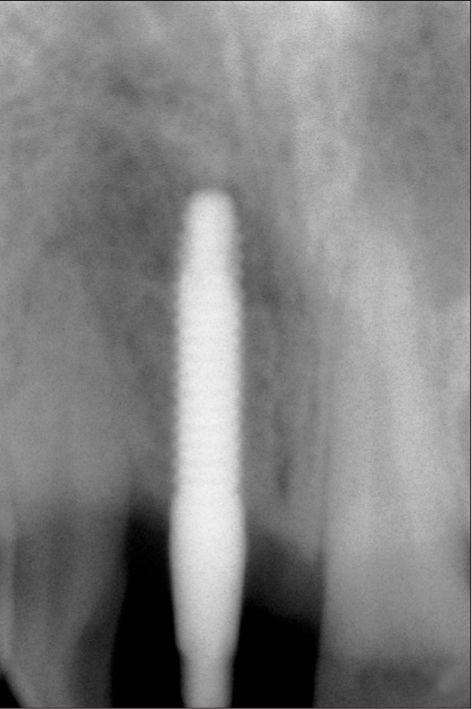
Immediate postoperative radiograph

**Figure 4c:**
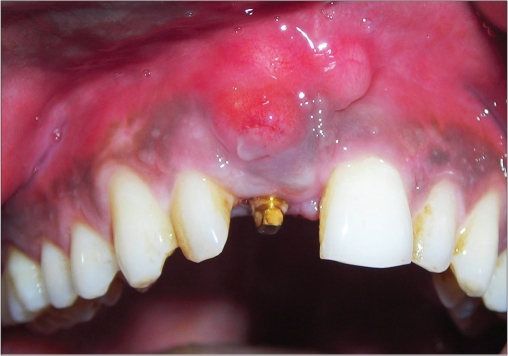
Two week photograph

**Figure 4d:**
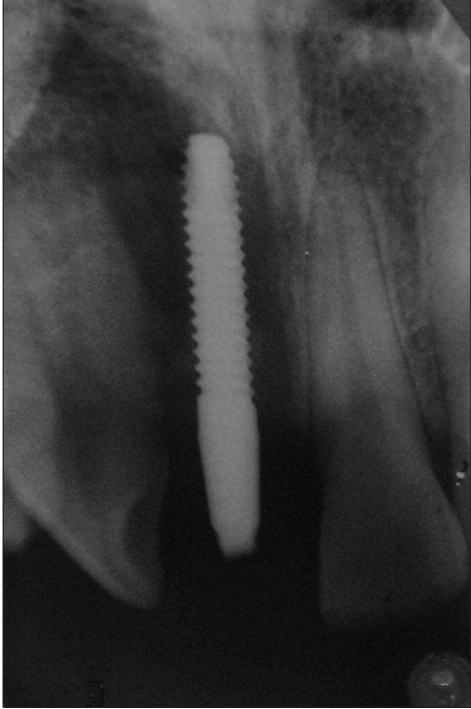
Two week radiograph

**Figure 4e:**
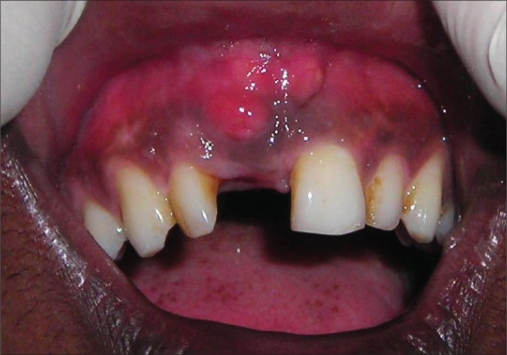
Three week photograph

**Figure 4f:**
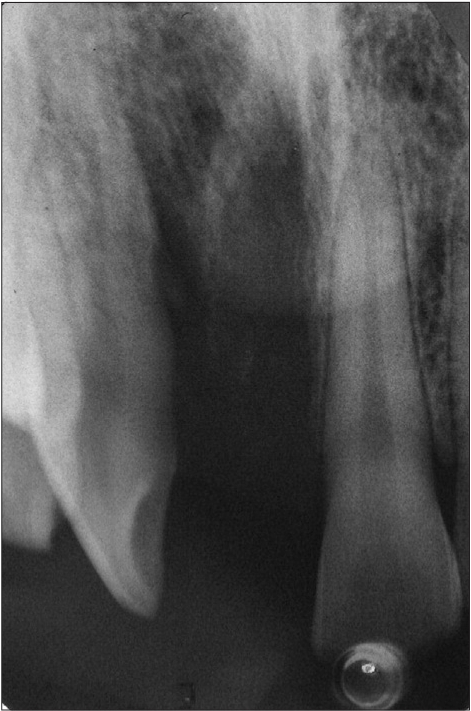
Three week radiograph

A 25-year-old male patient presented with a history of failed endodontic therapy in relation to tooth #11 and subsequent extraction 6 months prior to presentation to implant clinic. Clinical and radiologic examination revealed adequate hard and soft tissue dimensions for implant placement. A 3.0 × 15 mm single piece implant^$^ was placed in relation to #11 with good primary stability. In two weeks’ time patient presented with signs of peri-implant mucositis and mobility. The implant was removed the subsequent week.

#### Discussion

The existence of a periapical pathology necessitated extraction of the tooth after failure of endodontic therapy. Although there was no radiographic evidence of any pathology in the periapical region at the site of implant placement, the previous periapical pathology[16,17] had obviously not resolved. These sites may be considered to be at greater risk for implant placement and underlies the value of evaluation of the suspected sites with more sensitive investigative procedures such as CT scan. Economic considerations may preclude the use of such procedure routinely, but the suspected sites have to be evaluated with CT scan to avoid such retrograde implant failures.

## CONCLUSION

Retrograde peri-implantits constitutes an important source of implant failure. Careful preoperative evaluation of the site, adjacent teeth, and postoperative assessment of the implant placed could reduce the chances of development of retrograde peri-implantitis. Once diagnosed, the lesion has to be treated aggressively rather than by observation and conservative management.

^$^ BioHorizons Maximus^®^ Single Piece Implant

^$$^ Universal Implant Deplaquer (Straumann)

^#^ Regen Biocement and Hydrase (Steiner Laboratories)

^##^ Grabio Glascera

* Healiguide membrane
